# Crystal structure of 2-[(*E*)-2-(4-bromo­phen­yl)diazen-1-yl]-4,5-bis­(4-meth­oxy­phen­yl)-1*H*-imidazole: the first example of a structurally characterized tri­aryl­azo­imid­azole

**DOI:** 10.1107/S2056989021002024

**Published:** 2021-02-26

**Authors:** Ayalew Temesgen, Alexander G. Tskhovrebov, Anna V. Vologzhanina, Tuan A. Le, Victor N. Khrustalev

**Affiliations:** a Chemistry Department, College of Natural and Computational Sciences, University of Gondar, 196 Gondar, Ethiopia; b N.N. Semenov Federal Research Center for Chemical Physics, Russian Academy of Sciences, Ul. Kosygina 4, Moscow, Russian Federation; c Peoples’ Friendship University of Russia, 6 Miklukho-Maklaya Street, Moscow, 117198, Russian Federation; d Nesmeyanov Institute of Organoelement Compounds, Russian Academy of Sciences, Moscow, Vavilova str., 28, Russian Federation; e Faculty of Chemistry, VNU University of Science, Vietnam National University, Hanoi, 334 Nguyen Trai, Hanoi, 100000, Vietnam

**Keywords:** crystal structure, azo­imidazoles, nitro­gen heterocycles, dyes, *PASS* program

## Abstract

The title compound, C_23_H_19_BrN_4_O_2_, is a product of an azo coupling reaction between 3,4-bis­(4-meth­oxy­phen­yl)imidazole and 4-bromo­phenyl­diazo­nium tetra­fluoro­borate. Its crystal structure was determined using data collected at 120 K. The mol­ecule adopts a *trans* configuration with respect to the N=N double bond. The imidazole and aryl rings attached to the azo linkage are coplanar within 12.73 (14)°, which indicates significant electron delocalization within the mol­ecule. In the crystal, the mol­ecules form centrosymmetric dimers *via* pairs of N—H⋯O hydrogen bonds.

## Abstract 2

The mol­ecule of the title compound adopts a *trans* configuration with respect to the azo double bond.

## Chemical context   

Azo­imidazoles are a class of dyes that have found widespread applications in industry, as well as in laboratory research (Eymann *et al.*, 2016 ▸; Tskhovrebov *et al.*, 2014 ▸; Liu *et al.*, 2019 ▸). They are widely used for dyeing natural and synthetic fibers. In addition, they have found applications as photoswitches and hold promise for utilization in photopharmacology (Crespi *et al.*, 2019 ▸). Azo-functionalized imidazoles have been studied intensively as ligands in coordination chemistry (Sarker, Chand *et al.*, 2007 ▸; Sarker, Sardar *et al.*, 2007 ▸; Schütt *et al.*, 2016 ▸; Das *et al.*, 1997 ▸; Misra *et al.*, 1997 ▸). They are also attractive as chelating bidentate ligands. Azo­imidazole coordination compounds have been reported for numerous metals, some of them showing inter­esting photochromic properties (Sarker, Sardar *et al.*, 2007 ▸; Sarker, Chand *et al.*, 2007 ▸; Crespi *et al.*, 2019 ▸). Numerous publications have been devoted to the development of organic crystalline materials that contain various imidazole-based architectures (Akhriff *et al.*, 2006 ▸). Following our inter­est in azo dyes (Nenajdenko *et al.*, 2020 ▸; Tskhovrebov, Vasileva *et al.*, 2018 ▸), imidazole chemistry, imidazolylidenes and corresponding metal–carbene com­plexes (Tskhovrebov, Lingnau *et al.*, 2019 ▸; Tskhovrebov, Goddard *et al.*, 2018 ▸; Mikhaylov *et al.*, 2018 ▸; Tskhovrebov *et al.*, 2012 ▸), we report here the synthesis and crystal structure of (*E*)-2-[(4-bromo­phen­yl)diazen­yl]-4,5-bis­(4-meth­oxy­phen­yl)-1*H*-imidazole. Although azo­imidazoles form a widely studied class of azo compounds, tri­aryl­azo­imidazoles have never been structurally characterized. This work presents the first example of structurally characterized tri­aryl­azo­imidazole.
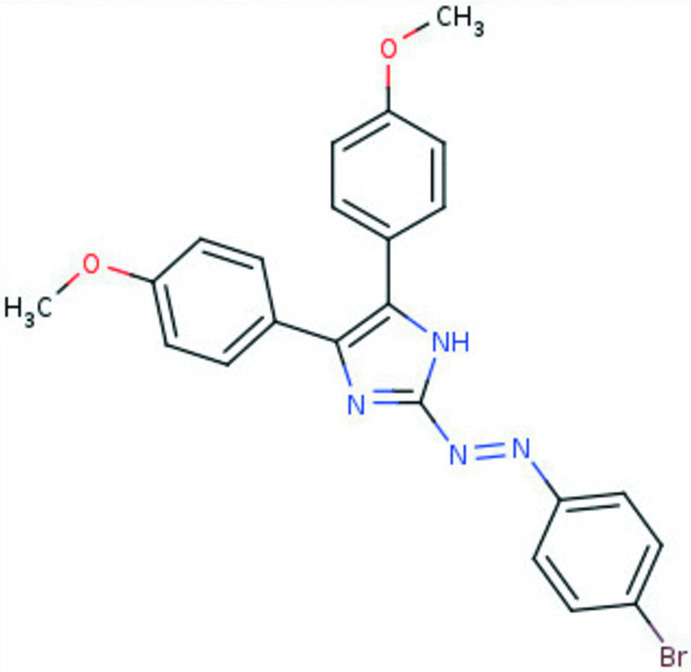


The *PASS* program (Filimonov *et al.*, 2014 ▸) predicted the potential activity of the title compound as a thiol protease inhibitor and an aspulvinone di­methyl­allyl­transferase inhib­itor at 81% and 76% probability levels, respectively.

## Structural commentary   

**Figure 1:**
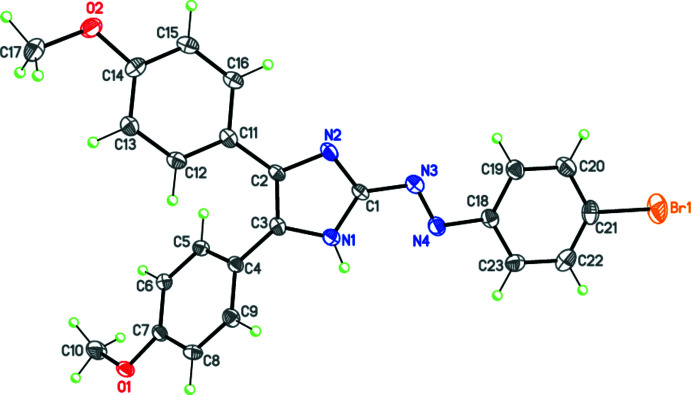
Mol­ecular structure of the title compound. Displacement ellipsoids are shown at the 50% probability level. The hydrogen atoms are presented as small spheres of arbitrary radius.

The mol­ecular structure of the title compound is shown in Fig. 1 ▸. Overall, bond dimensions within the mol­ecule are similar to those reported for structurally relevant azo compounds (Tskhovrebov *et al.*, 2014 ▸, 2015 ▸; Liu *et al.*, 2019 ▸; Eymann *et al.*, 2016 ▸; Nenajdenko *et al.*, 2020 ▸). The mol­ecule adopts a *trans* configuration with respect to the azo double bond. The N=N bond distance of 1.274 (3) Å is slightly longer than that in azo­benzene. The imidazole and aryl rings attached to the azo group are coplanar within 12.73 (14)°, which indicates significant electron delocalization within the mol­ecule. The two other aromatic rings, C4–C9 and C11–C16, form dihedral angles with the plane of the imidazole ring of 60.64 (14) and 22.38 (13)°, respectively.

## Supra­molecular features   

**Figure 2:**
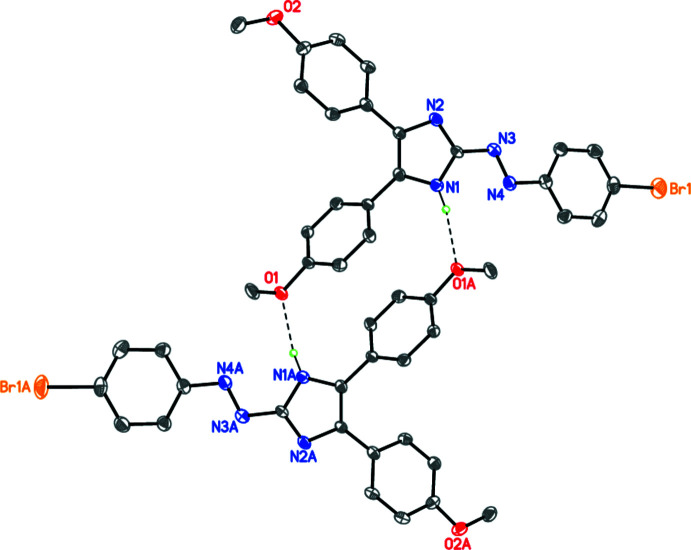
The hydrogen-bonded centrosymmetric dimer. Dashed lines indicate the N—H⋯O hydrogen bonds.

**Figure 3:**
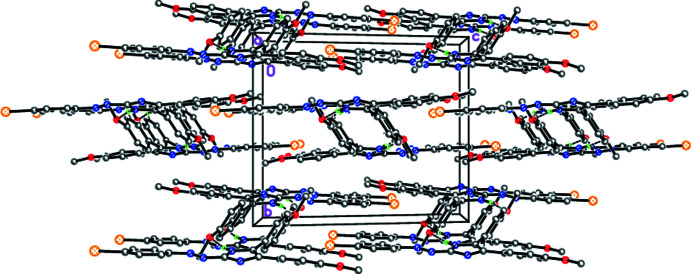
Crystal packing projected along the-*a* axis direction.

In the crystal, the title mol­ecules form centrosymmetric dimers *via* pairs of N—H⋯O hydrogen bonds (Fig. 2 ▸, Table 1 ▸). A similar supra­molecular motif has previously been observed by our group (Repina *et al.*, 2020 ▸; Tskhovrebov, Novikov *et al.*, 2019 ▸). The crystal packing involves some π–π stacking inter­actions (Fig. 3 ▸) with a shortest inter­centroid separation of 3.792 (2) Å between two imidazole rings related by the symmetry operation 1 − *x*, 1 − *y*, 1 − *z*.

## Database survey   

A search of the Cambridge Structural Database (CSD version 5.41, update of March 2020; Groom *et al.*, 2016 ▸) revealed that this is the first example of a structurally characterized tri­aryl­azomidazole. At the same time, the CSD search revealed several examples of structurally similar azo­imidazoles, which contain a proton at the imidazolic N atom, *viz*. 2-[(4-bromo­phen­yl)diazen­yl]-1*H*-imidazole (Pramanik *et al.*, 2010 ▸), 2-(1-naphthyl­diazen­yl)-1*H*-imidazole (Pramanik *et al.*, 2010 ▸), 2-[4-(*N*,*N*-di­hydroxy­ethyl­amino)­phenyl­azo]-4,5-di­cyano­imidazole (Carella *et al.*, 2004 ▸), phenyl­azo­imidazole (Fun *et al.*, 1999 ▸), 4-(4,5-di­cyano-1*H*-imidazolyazo)-*N*,*N*-di­ethyl­aniline (Zhang *et al.*, 2007 ▸), 2-(*p*-tolyl­azo)imidazole (Bhunia *et al.*, 2006 ▸) and 3,3′-({4-[(4,5-di­cyano-1*H*-imidazol-2-yl)diazen­yl]phen­yl}im­ino) dipropionic acid (Centore *et al.*, 2013 ▸).

## Synthesis and crystallization   

Tri­aryl­azo­imidazole was prepared according to the literature method (Fun *et al.*, 1999 ▸) *via* azo coupling of *p*-bromo­phenyl­diazo­nium tetra­fluoro­borate with di(*p*-anis­yl)imidazole and isolated in 84% yield as a red solid. Crystals suitable for X-ray analysis were obtained by slow evaporation of a saturated MeOH solution.

## Refinement   

Crystal data, details of data collection, and results of structure refinement are summarized in Table 2 ▸. The X-ray diffraction study was performed using the equipment of the Center for Mol­ecular Studies of INEOS RAS. The hydrogen atom of the NH group was localized in the difference-Fourier map and refined with a fixed isotropic displacement parameter [*U*_iso_(H) = 1.2*U*_eq_(N)]. The other hydrogen atoms were placed in calculated positions with C—H = 0.95–0.98 Å and refined using a riding model with fixed isotropic displacement parameters [*U*_iso_(H) = 1.5*U*_eq_(C) for CH_3_ groups and *U*_iso_(H) = 1.2*U*_eq_(C) for other groups].

## Supplementary Material

Crystal structure: contains datablock(s) I. DOI: 10.1107/S2056989021002024/yk2143sup1.cif

Structure factors: contains datablock(s) I. DOI: 10.1107/S2056989021002024/yk2143Isup2.hkl

Supporting information file. DOI: 10.1107/S2056989021002024/yk2143Isup3.cml

CCDC reference: 2064019

Additional supporting information:  crystallographic information; 3D view; checkCIF report

## Figures and Tables

**Table 1:** Hydrogen-bond geometry (Å, °)

*D*—H⋯*A*	*D*—H	H⋯*A*	*D*⋯*A*	*D*—H⋯*A*
N1—H1⋯O1^i^	0.80 (3)	2.17 (3)	2.963 (3)	169 (3)

Symmetry code: (i) -x, -y+1, -z+1.

**Table 2:** Experimental details

Crystal data	
Chemical formula	C_23_H_19_BrN_4_O_2_
*M*_r_	463.32
Crystal system, space group	Monoclinic, *P*2_1_/*c*
Temperature (K)	120
*a*, *b*, *c* (Å)	10.7812 (9), 12.7877 (11), 15.4575 (13)
β (°)	109.635 (2)
*V* (Å^3^)	2007.2 (3)
*Z*	4
Radiation type	Mo *K*α
μ (mm^−1^)	2.08
Crystal size (mm)	0.33 × 0.21 × 0.08

Data collection	
Diffractometer	Bruker APEXII CCD
Absorption correction	Multi-scan (*SADABS*; Krause *et al.*, 2015 ▸)
*T*_min_, *T*_max_	0.597, 0.746
No. of measured, independent and observed [*I* > 2σ(*I*)] reflections	22147, 6069, 3449
*R*_int_	0.084
(sin θ/λ)_max_ (Å^−1^)	0.714

Refinement	
*R*[*F* ^2^ > 2σ(*F* ^2^)], *wR*(*F* ^2^), *S*	0.052, 0.122, 1.00
No. of reflections	6069
No. of parameters	276
H-atom treatment	H atoms treated by a mixture of independent and constrained refinement
Δρ_max_, Δρ_min_ (e Å^−3^)	0.44, −0.62

Computer programs: *APEX3* (Bruker, 2018 ▸), *SAINT* (Bruker, 2013 ▸), *SHELXT* (Sheldrick, 2015*a* ▸), *SHELXL* (Sheldrick, 2015*b* ▸) and *SHELXTL* (Sheldrick, 2008 ▸).

## supplementary crystallographic information

### Crystal data

**Table:**

C_23_H_19_BrN_4_O_2_	*F*(000) = 944
*M**_r_* = 463.32	*D*_x_ = 1.533 Mg m^−^^3^
Monoclinic, *P*2_1_/*c*	Mo *K*α radiation, λ = 0.71073 Å
*a* = 10.7812 (9) Å	Cell parameters from 2288 reflections
*b* = 12.7877 (11) Å	θ = 2.6–25.4°
*c* = 15.4575 (13) Å	µ = 2.08 mm^−^^1^
β = 109.635 (2)°	*T* = 120 K
*V* = 2007.2 (3) Å^3^	Plate, orange
*Z* = 4	0.33 × 0.21 × 0.08 mm

### Data collection

**Table:**

Bruker APEXII CCD diffractometer	3449 reflections with *I* > 2σ(*I*)
φ and ω scans	*R*_int_ = 0.084
Absorption correction: multi-scan (SADABS; Krause *et al.*, 2015)	θ_max_ = 30.5°, θ_min_ = 2.0°
*T*_min_ = 0.597, *T*_max_ = 0.746	*h* = −14→15
22147 measured reflections	*k* = −18→18
6069 independent reflections	*l* = −21→21

### Refinement

**Table:**

Refinement on *F*^2^	Primary atom site location: difference Fourier map
Least-squares matrix: full	Secondary atom site location: difference Fourier map
*R*[*F*^2^ > 2σ(*F*^2^)] = 0.052	Hydrogen site location: mixed
*wR*(*F*^2^) = 0.122	H atoms treated by a mixture of independent and constrained refinement
*S* = 1.00	*w* = 1/[σ^2^(*F*_o_^2^) + (0.0498*P*)^2^] where *P* = (*F*_o_^2^ + 2*F*_c_^2^)/3
6069 reflections	(Δ/σ)_max_ < 0.001
276 parameters	Δρ_max_ = 0.44 e Å^−^^3^
0 restraints	Δρ_min_ = −0.61 e Å^−^^3^

### Special details

**Table:**

Experimental. SADABS-2014/5 (Bruker, 2014/5) was used for absorption correction. wR2(int) was 0.0815 before and 0.0536 after correction. The Ratio of minimum to maximum transmission is 0.8000. The λ/2 correction factor is 0.00150.
Geometry. All esds (except the esd in the dihedral angle between two l.s. planes) are estimated using the full covariance matrix. The cell esds are taken into account individually in the estimation of esds in distances, angles and torsion angles; correlations between esds in cell parameters are only used when they are defined by crystal symmetry. An approximate (isotropic) treatment of cell esds is used for estimating esds involving l.s. planes.

### Fractional atomic coordinates and isotropic or equivalent isotropic displacement parameters (Å^2^)

**Table:**

	*x*	*y*	*z*	*U*_iso_*/*U*_eq_	
Br1	0.27445 (4)	0.40711 (3)	−0.14822 (2)	0.04431 (14)	
O1	−0.01642 (18)	0.55364 (14)	0.68807 (12)	0.0212 (4)	
O2	0.84035 (18)	0.33604 (14)	0.90163 (12)	0.0249 (4)	
N1	0.2914 (2)	0.39235 (17)	0.42224 (15)	0.0191 (5)	
H1	0.222 (3)	0.410 (2)	0.387 (2)	0.023*	
N2	0.5047 (2)	0.35277 (17)	0.47136 (14)	0.0194 (5)	
N3	0.4041 (2)	0.36812 (17)	0.30966 (15)	0.0204 (5)	
N4	0.2927 (2)	0.38470 (16)	0.24827 (15)	0.0215 (5)	
C1	0.3985 (3)	0.36862 (19)	0.39831 (17)	0.0176 (5)	
C2	0.4641 (3)	0.37005 (19)	0.54552 (17)	0.0162 (5)	
C3	0.3314 (3)	0.39603 (19)	0.51634 (17)	0.0178 (5)	
C4	0.2423 (3)	0.43346 (19)	0.56408 (17)	0.0163 (5)	
C5	0.2757 (3)	0.5239 (2)	0.61663 (17)	0.0192 (6)	
H5	0.3570	0.5577	0.6235	0.023*	
C6	0.1921 (3)	0.56603 (19)	0.65933 (17)	0.0177 (6)	
H6	0.2161	0.6281	0.6949	0.021*	
C7	0.0742 (3)	0.51714 (19)	0.64976 (17)	0.0175 (5)	
C8	0.0393 (3)	0.4255 (2)	0.59788 (18)	0.0203 (6)	
H8	−0.0416	0.3915	0.5918	0.024*	
C9	0.1230 (3)	0.38447 (19)	0.55551 (17)	0.0190 (6)	
H9	0.0990	0.3223	0.5202	0.023*	
C10	0.0165 (3)	0.6508 (2)	0.7369 (2)	0.0321 (7)	
H10A	0.0296	0.7048	0.6958	0.048*	
H10B	0.0978	0.6422	0.7895	0.048*	
H10C	−0.0552	0.6717	0.7588	0.048*	
C11	0.5584 (3)	0.36163 (19)	0.64007 (17)	0.0175 (5)	
C12	0.5186 (3)	0.34390 (19)	0.71556 (18)	0.0195 (6)	
H12	0.4273	0.3382	0.7065	0.023*	
C13	0.6090 (3)	0.3342 (2)	0.80404 (18)	0.0201 (6)	
H13	0.5800	0.3203	0.8545	0.024*	
C14	0.7421 (3)	0.34526 (19)	0.81732 (17)	0.0199 (6)	
C15	0.7843 (3)	0.3655 (2)	0.74336 (18)	0.0208 (6)	
H15	0.8753	0.3754	0.7531	0.025*	
C16	0.6935 (3)	0.3714 (2)	0.65562 (18)	0.0200 (6)	
H16	0.7232	0.3822	0.6050	0.024*	
C17	0.7997 (3)	0.3123 (2)	0.97884 (18)	0.0289 (7)	
H17A	0.7433	0.2501	0.9652	0.043*	
H17B	0.7506	0.3716	0.9911	0.043*	
H17C	0.8775	0.2989	1.0329	0.043*	
C18	0.2974 (3)	0.38963 (19)	0.15723 (18)	0.0198 (6)	
C19	0.4131 (3)	0.3895 (2)	0.13582 (18)	0.0234 (6)	
H19	0.4961	0.3872	0.1835	0.028*	
C20	0.4069 (3)	0.3928 (2)	0.04512 (19)	0.0270 (7)	
H20	0.4851	0.3907	0.0298	0.032*	
C21	0.2842 (3)	0.3992 (2)	−0.02354 (18)	0.0272 (7)	
C22	0.1682 (3)	0.4025 (2)	−0.0033 (2)	0.0294 (7)	
H22	0.0853	0.4075	−0.0508	0.035*	
C23	0.1765 (3)	0.3983 (2)	0.08831 (19)	0.0250 (6)	
H23	0.0984	0.4014	0.1037	0.030*	

### Atomic displacement parameters (Å^2^)

**Table:**

	*U*^11^	*U*^22^	*U*^33^	*U*^12^	*U*^13^	*U*^23^
Br1	0.0599 (3)	0.0551 (2)	0.01949 (15)	0.01172 (18)	0.01531 (15)	0.00843 (14)
O1	0.0173 (10)	0.0258 (10)	0.0236 (10)	−0.0033 (8)	0.0110 (8)	−0.0072 (8)
O2	0.0192 (11)	0.0302 (11)	0.0220 (10)	−0.0016 (8)	0.0025 (8)	0.0041 (8)
N1	0.0171 (12)	0.0223 (12)	0.0187 (11)	0.0021 (10)	0.0073 (10)	0.0012 (9)
N2	0.0197 (13)	0.0231 (12)	0.0192 (11)	0.0024 (9)	0.0116 (10)	0.0030 (9)
N3	0.0207 (13)	0.0200 (11)	0.0223 (11)	0.0010 (9)	0.0098 (10)	0.0001 (9)
N4	0.0229 (13)	0.0228 (12)	0.0212 (11)	−0.0008 (9)	0.0105 (10)	−0.0023 (9)
C1	0.0159 (14)	0.0201 (13)	0.0200 (13)	0.0009 (10)	0.0103 (11)	−0.0004 (10)
C2	0.0165 (14)	0.0168 (12)	0.0180 (12)	−0.0010 (10)	0.0096 (11)	0.0015 (9)
C3	0.0200 (14)	0.0182 (13)	0.0170 (12)	0.0008 (11)	0.0089 (11)	0.0011 (10)
C4	0.0156 (14)	0.0189 (13)	0.0157 (12)	0.0033 (10)	0.0068 (11)	0.0025 (9)
C5	0.0158 (14)	0.0244 (14)	0.0180 (12)	−0.0027 (11)	0.0062 (11)	0.0012 (10)
C6	0.0177 (14)	0.0189 (13)	0.0167 (12)	−0.0004 (10)	0.0062 (11)	−0.0008 (9)
C7	0.0159 (14)	0.0224 (14)	0.0162 (12)	0.0029 (11)	0.0080 (11)	0.0024 (10)
C8	0.0159 (14)	0.0224 (14)	0.0235 (13)	−0.0059 (10)	0.0080 (11)	−0.0009 (10)
C9	0.0214 (15)	0.0171 (14)	0.0204 (13)	0.0001 (10)	0.0095 (12)	−0.0023 (10)
C10	0.0271 (18)	0.0387 (18)	0.0361 (18)	−0.0066 (14)	0.0181 (15)	−0.0197 (14)
C11	0.0213 (15)	0.0138 (12)	0.0204 (13)	0.0017 (10)	0.0108 (11)	0.0014 (10)
C12	0.0167 (14)	0.0206 (14)	0.0228 (13)	0.0000 (11)	0.0087 (11)	0.0022 (10)
C13	0.0216 (15)	0.0205 (14)	0.0200 (13)	0.0005 (11)	0.0094 (12)	0.0015 (10)
C14	0.0197 (15)	0.0154 (13)	0.0210 (13)	−0.0009 (10)	0.0021 (11)	0.0008 (10)
C15	0.0176 (15)	0.0185 (13)	0.0289 (14)	0.0020 (11)	0.0112 (12)	0.0030 (11)
C16	0.0179 (15)	0.0199 (13)	0.0251 (14)	0.0019 (11)	0.0112 (12)	0.0025 (10)
C17	0.0272 (17)	0.0354 (17)	0.0201 (14)	−0.0006 (13)	0.0027 (13)	0.0011 (12)
C18	0.0239 (16)	0.0168 (13)	0.0215 (13)	−0.0027 (11)	0.0113 (12)	0.0003 (10)
C19	0.0246 (16)	0.0261 (15)	0.0205 (13)	0.0041 (12)	0.0087 (12)	0.0030 (11)
C20	0.0264 (17)	0.0315 (16)	0.0271 (15)	0.0039 (13)	0.0141 (13)	0.0044 (12)
C21	0.0401 (19)	0.0242 (15)	0.0184 (13)	0.0012 (13)	0.0113 (13)	0.0024 (11)
C22	0.0322 (18)	0.0274 (16)	0.0235 (14)	−0.0054 (13)	0.0028 (13)	−0.0007 (12)
C23	0.0203 (15)	0.0297 (16)	0.0245 (14)	−0.0048 (12)	0.0069 (12)	−0.0008 (12)

### Geometric parameters (Å, º)

**Table:**

Br1—C21	1.897 (3)	C10—H10A	0.9800
O1—C7	1.382 (3)	C10—H10B	0.9800
O1—C10	1.435 (3)	C10—H10C	0.9800
O2—C14	1.381 (3)	C11—C12	1.390 (4)
O2—C17	1.435 (3)	C11—C16	1.400 (4)
N1—C1	1.360 (3)	C12—C13	1.393 (4)
N1—C3	1.372 (3)	C12—H12	0.9500
N1—H1	0.80 (3)	C13—C14	1.387 (4)
N2—C1	1.326 (3)	C13—H13	0.9500
N2—C2	1.375 (3)	C14—C15	1.389 (4)
N3—N4	1.274 (3)	C15—C16	1.382 (4)
N3—C1	1.392 (3)	C15—H15	0.9500
N4—C18	1.427 (3)	C16—H16	0.9500
C2—C3	1.388 (4)	C17—H17A	0.9800
C2—C11	1.477 (4)	C17—H17B	0.9800
C3—C4	1.474 (4)	C17—H17C	0.9800
C4—C5	1.390 (3)	C18—C23	1.383 (4)
C4—C9	1.397 (4)	C18—C19	1.393 (4)
C5—C6	1.392 (4)	C19—C20	1.382 (4)
C5—H5	0.9500	C19—H19	0.9500
C6—C7	1.379 (4)	C20—C21	1.392 (4)
C6—H6	0.9500	C20—H20	0.9500
C7—C8	1.399 (3)	C21—C22	1.388 (5)
C8—C9	1.384 (4)	C22—C23	1.389 (4)
C8—H8	0.9500	C22—H22	0.9500
C9—H9	0.9500	C23—H23	0.9500
			
C7—O1—C10	115.6 (2)	C12—C11—C2	122.6 (2)
C14—O2—C17	116.8 (2)	C16—C11—C2	119.6 (2)
C1—N1—C3	107.6 (2)	C11—C12—C13	121.8 (3)
C1—N1—H1	125 (2)	C11—C12—H12	119.1
C3—N1—H1	127 (2)	C13—C12—H12	119.1
C1—N2—C2	105.0 (2)	C14—C13—C12	119.0 (2)
N4—N3—C1	113.0 (2)	C14—C13—H13	120.5
N3—N4—C18	113.9 (2)	C12—C13—H13	120.5
N2—C1—N1	111.8 (2)	O2—C14—C13	123.9 (2)
N2—C1—N3	121.8 (2)	O2—C14—C15	115.7 (2)
N1—C1—N3	126.2 (2)	C13—C14—C15	120.4 (2)
N2—C2—C3	110.5 (2)	C16—C15—C14	119.9 (3)
N2—C2—C11	120.5 (2)	C16—C15—H15	120.1
C3—C2—C11	129.1 (2)	C14—C15—H15	120.1
N1—C3—C2	105.0 (2)	C15—C16—C11	121.1 (2)
N1—C3—C4	121.1 (2)	C15—C16—H16	119.4
C2—C3—C4	133.5 (2)	C11—C16—H16	119.4
C5—C4—C9	118.6 (2)	O2—C17—H17A	109.5
C5—C4—C3	118.6 (2)	O2—C17—H17B	109.5
C9—C4—C3	122.8 (2)	H17A—C17—H17B	109.5
C4—C5—C6	121.2 (3)	O2—C17—H17C	109.5
C4—C5—H5	119.4	H17A—C17—H17C	109.5
C6—C5—H5	119.4	H17B—C17—H17C	109.5
C7—C6—C5	119.6 (2)	C23—C18—C19	120.3 (2)
C7—C6—H6	120.2	C23—C18—N4	115.2 (2)
C5—C6—H6	120.2	C19—C18—N4	124.4 (2)
C6—C7—O1	124.0 (2)	C20—C19—C18	119.9 (3)
C6—C7—C8	120.1 (2)	C20—C19—H19	120.0
O1—C7—C8	115.9 (2)	C18—C19—H19	120.0
C9—C8—C7	119.8 (2)	C19—C20—C21	119.0 (3)
C9—C8—H8	120.1	C19—C20—H20	120.5
C7—C8—H8	120.1	C21—C20—H20	120.5
C8—C9—C4	120.8 (2)	C22—C21—C20	121.8 (3)
C8—C9—H9	119.6	C22—C21—Br1	118.8 (2)
C4—C9—H9	119.6	C20—C21—Br1	119.4 (2)
O1—C10—H10A	109.5	C21—C22—C23	118.3 (3)
O1—C10—H10B	109.5	C21—C22—H22	120.8
H10A—C10—H10B	109.5	C23—C22—H22	120.8
O1—C10—H10C	109.5	C18—C23—C22	120.6 (3)
H10A—C10—H10C	109.5	C18—C23—H23	119.7
H10B—C10—H10C	109.5	C22—C23—H23	119.7
C12—C11—C16	117.8 (2)		
			
C1—N3—N4—C18	177.1 (2)	C3—C4—C9—C8	−176.7 (2)
C2—N2—C1—N1	−1.7 (3)	N2—C2—C11—C12	−158.2 (2)
C2—N2—C1—N3	173.7 (2)	C3—C2—C11—C12	22.9 (4)
C3—N1—C1—N2	2.4 (3)	N2—C2—C11—C16	21.6 (3)
C3—N1—C1—N3	−172.7 (2)	C3—C2—C11—C16	−157.2 (3)
N4—N3—C1—N2	180.0 (2)	C16—C11—C12—C13	−1.1 (4)
N4—N3—C1—N1	−5.3 (4)	C2—C11—C12—C13	178.8 (2)
C1—N2—C2—C3	0.4 (3)	C11—C12—C13—C14	1.7 (4)
C1—N2—C2—C11	−178.7 (2)	C17—O2—C14—C13	0.8 (4)
C1—N1—C3—C2	−2.0 (3)	C17—O2—C14—C15	−178.4 (2)
C1—N1—C3—C4	171.9 (2)	C12—C13—C14—O2	−179.2 (2)
N2—C2—C3—N1	1.0 (3)	C12—C13—C14—C15	−0.1 (4)
C11—C2—C3—N1	180.0 (2)	O2—C14—C15—C16	177.1 (2)
N2—C2—C3—C4	−171.7 (3)	C13—C14—C15—C16	−2.1 (4)
C11—C2—C3—C4	7.2 (5)	C14—C15—C16—C11	2.7 (4)
N1—C3—C4—C5	−115.9 (3)	C12—C11—C16—C15	−1.2 (4)
C2—C3—C4—C5	55.9 (4)	C2—C11—C16—C15	179.0 (2)
N1—C3—C4—C9	61.2 (3)	N3—N4—C18—C23	174.5 (2)
C2—C3—C4—C9	−127.0 (3)	N3—N4—C18—C19	−7.5 (4)
C9—C4—C5—C6	−0.6 (4)	C23—C18—C19—C20	−3.2 (4)
C3—C4—C5—C6	176.6 (2)	N4—C18—C19—C20	178.9 (2)
C4—C5—C6—C7	0.2 (4)	C18—C19—C20—C21	1.8 (4)
C5—C6—C7—O1	−178.9 (2)	C19—C20—C21—C22	0.1 (4)
C5—C6—C7—C8	0.4 (4)	C19—C20—C21—Br1	178.5 (2)
C10—O1—C7—C6	2.6 (4)	C20—C21—C22—C23	−0.6 (4)
C10—O1—C7—C8	−176.7 (2)	Br1—C21—C22—C23	−179.0 (2)
C6—C7—C8—C9	−0.6 (4)	C19—C18—C23—C22	2.8 (4)
O1—C7—C8—C9	178.7 (2)	N4—C18—C23—C22	−179.2 (2)
C7—C8—C9—C4	0.1 (4)	C21—C22—C23—C18	−0.8 (4)
C5—C4—C9—C8	0.4 (4)		

### Hydrogen-bond geometry (Å, º)

**Table:**

*D*—H···*A*	*D*—H	H···*A*	*D*···*A*	*D*—H···*A*
N1—H1···O1^i^	0.80 (3)	2.17 (3)	2.963 (3)	169 (3)

Symmetry code: (i) −*x*, −*y*+1, −*z*+1.
